# Efficient and Durable Sodium, Chloride‐doped Iron Oxide‐Hydroxide Nanohybrid‐Promoted Capacitive Deionization of Saline Water via Synergetic Pseudocapacitive Process

**DOI:** 10.1002/advs.202201678

**Published:** 2022-07-11

**Authors:** Jingxuan Zhao, Bingyao Wu, Xinwei Huang, Yang Sun, Zhibo Zhao, Meidan Ye, Xiaoru Wen

**Affiliations:** ^1^ College of Chemistry and Chemical Engineering Inner Mongolia University Hohhot 010021 P. R. China; ^2^ Research Institute for Biomimetics and Soft Matter Fujian Provincial Key Laboratory for Soft Functional Materials Research Department of Physics College of Physical Science and Technology Xiamen University Xiamen 361005 P. R. China

**Keywords:** capacitive deionization, doping engineering, hierarchical nanopore alignment, iron oxide‐hydroxide, synergetic pseudocapacitive removal mechanism

## Abstract

Recently, the rational design and development of efficient faradaic deionization electrodes with high theoretical capacitance, natural abundance, and attractive conductivity have shown great promise for outstanding capacitive deionization (CDI)‐based desalination applications. Herein, the construction of novel FeOOH hybrid heterostructures with Na and Cl dopants (e.g., Na‐FeOOH and Cl‐FeOOH) via a robust hydrothermal strategy is reported, and an asymmetric CDI cell (Na‐FeOOH//Cl‐FeOOH) comprising Na‐FeOOH and Cl‐FeOOH working as the cathode and anode, respectively, is assembled. The multiple coupling effects of the specific structural features (e.g., enriched porosity, hierarchical pore alignment, and highly open crystalline framework), enhanced electrochemical conductivity, and optimized ion‐transfer property endow the FeOOH hybrid electrode with improved electrochemical performance. Impressively, the Na‐FeOOH//Cl‐FeOOH cell demonstrates a superior salt adsorption capacity (SAC_NaCl_) of 35.12 mg g^−1^ in a 500 mg L^−1^ NaCl solution, a faster removal rate, and remarkable cycling stability. Moreover, the pseudocapacitive removal mechanism from the synergetic contribution of the Na‐FeOOH cathode and Cl‐FeOOH anode account for the significant desalination promotion of the Na‐FeOOH//Cl‐FeOOH cell.

## Graphical abstract

Novel Na, Cl‐doped FeOOH nanohybrids (Na‐FeOOH and Cl‐FeOOH) are constructed via a robust hydrothermal strategy. The asymmetric Na‐FeOOH//Cl‐FeOOH capacitive deionization (CDI) cell demonstrates a superior salt adsorption capacity (SAC_NaCl_) of 35.12 mg g^−1^ in a 500 mg L^−1^ NaCl solution and remarkable cycling stability. The synergetic pseudocapacitive contribution from Na‐FeOOH cathode and Cl‐FeOOH anode leads to the significant desalination promotion of the Na‐FeOOH//Cl‐FeOOH cell.

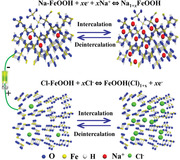

## 1 Introduction

Global water scarcity due to rapid population growth, ubiquitous industrial contamination, and uneven freshwater distribution has impelled the urgent development of efficient water purification technologies to meet the rapidly expanding demand for clean water.^[^ ^1^, ^2^, ^3^, ^4^, ^5^ ^]^ Capacitive deionization (CDI), a newly emerging low energy consumption electrochemical water treatment method that has high efficiency and environmental friendliness, has attracted considerable attention.^[^ ^3^, ^6^, ^7^, ^8^ ^]^ Considerable efforts have been devoted to updating cell configuration and optimizing and innovating electrode materials, indicating that the electrode materials play a crucial role in highly efficient CDI applications. These widely reported materials can be mainly divided into two types: 1) electrochemical double‐layer capacitive materials including graphene, activated carbon (AC), and carbon nanotubes;^[^ ^9^, ^10^, ^11^, ^12^ ^]^ 2) pseudocapacitive materials including metal oxides, sulfides, and hydroxides.^[^ ^6^, ^13^, ^14^, ^15^, ^16^ ^]^ Two unresolved issues still remain in traditional porous carbon electrodes: the unsatisfied desalination capacity and feasibility limitation of the highly concentrated saline water. Notably, the pioneering work in developing a faradaic electrode‐based asymmetric desalting battery by Pasta's group opened up a novel avenue toward boosting CDI performance.^[^ ^17^ ^]^ Various pseudocapacitive materials for Na‐ion capture based on redox reactions have been actively explored, and the corresponding electrodes show enhanced deionization capacity and remarkable stability in high‐concentration feedwater.^[^ ^18^, ^19^, ^20^, ^21^ ^]^ In particular, transition metal compound (TMC)‐based nanomaterials with dramatical chemical/physical superiorities (e.g., high theoretical capacitance, natural abundance, and attractive conductivity) have been extensively utilized as pseudocapacitive electrodes in energy storage systems (e.g., sodium‐ion batteries, supercapacitors, lithium‐ion batteries, and desalination cells).^[^ ^8^, ^22^, ^23^, ^24^ ^]^ Nevertheless, bare TMC‐based CDI electrodes, which have unsatisfactory desalination capacity and inferior durability, still need to be further optimized via material morphology/microstructure/composition regulation and doping/composite engineering. Pseudocapacitive electrode materials with superior electronic conductivity, excellent cyclic stability, and high capacity are also of great significance for the large‐scale practical application of CDI‐based saline purification techniques, but challenges still exist.

Among the TMC candidates, Fe‐based nanomaterials (e.g., Fe_2_O_3_, Fe_3_O_4_, and FeOOH), which have nontoxic characteristics, high theoretical specific capacitance, low cost, and open structural features, hold great potential for use in electrochemical storage and conversion devices. It is noteworthy that, FeOOH polymorphs mainly comprising the most common and stable goethite (*α*‐FeOOH), relatively stable akaganeite (*β*‐FeOOH), metastable lepidocrocite (*γ*‐FeOOH), and ferromagnetic feroxyhyte (*δ*‐FeOOH), the rising stars as pseudocapacitive alternatives, have been extensively investigated as electrode materials for batteries, supercapacitors, water splitting, and other applications.^[^ ^25^, ^26^, ^27^, ^28^, ^29^, ^30^, ^31^, ^32^, ^33^, ^34^ ^]^ However, compared to that of CoOOH and NiOOH, the wide development of Fe‐based pseudocapacitive electrode materials remains a significant challenge owing to the inherently poor electrical conductivity and inevitably degrading structural stability resulting from large volume expansion during the consecutive intercalation/deintercalation process. Heteroatom doping has attracted recent attention as a means of overcoming these drawbacks, and various metals (Mn, Co, Ni, and, K, etc.) and nonmetal dopants (Cl and F) have been successfully incorporated.^[^ ^35^, ^36^, ^37^, ^38^, ^39^, ^40^ ^]^ The advantages of proper lattice parameter modification, newly produced defects/vacancies, and tailored electronic properties that have been attributed to the strong synergistic effects between the different elements after rational doping, will improve the electrochemical performance of FeOOH hybrids with improved deionization capacity and cycling durability. However, to the best of our knowledge, the necessary research on the CDI investigation of FeOOH‐based nanomaterials has not been reported.

Furthermore, to realize high‐performance CDI application, intensive efforts have been focused on Na‐ion capture pseudocapacitive electrode materials especially sodium metal oxides/phosphates and various TMCs (e.g., sulfides, nitrides, carbides, and oxides) in the past few years, while little attention has been paid to Cl‐ion capture electrodes.^[^ ^14^, ^21^, ^41^, ^42^, ^43^, ^44^, ^45^, ^46^ ^]^ Recently, several amazing faradaic alternatives (e.g., AgCl, Bi NCs@CNBs, CoFeCl‐layered double hydroxide (LDH), and ZnCo‐Cl LDH) to the widely used AC electrodes have been investigated. These alternatives demonstrate prominent Cl ion removal behaviors owing to the efficient Cl ion exchange‐promoted redox conversion process.^[^ ^47^, ^48^, ^49^, ^50^ ^]^ These developments show that further exploitation of effective and novel electrode materials for Cl ion capture is in high demand, but remains a daunting challenge for researchers. Notably, the *β*‐phase FeOOH(Cl) derivatives with a Cl ion dopant partly occupied the free tunnel sites of bare FeOOH are believed to play an essential role in building and stabilizing its unique tunnel‐type nanopore framework.^[^ ^40^ ^]^ Enriched and freely accessible Cl ions endow FeOOH(Cl) with great promise for efficient Cl storage, but the CDI performance evaluation of the FeOOH(Cl)‐based electrode has not been investigated to date.

**Figure 1:**
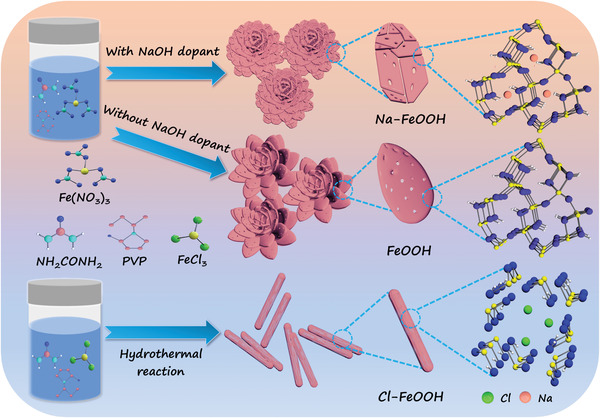
Schematic illustrations of preparation processing of Na‐FeOOH, Cl‐FeOOH, and bare FeOOH.

Inspired by these interesting studies, in this work, a novel asymmetric CDI cell (Na‐FeOOH//Cl‐FeOOH) consisting of Na‐doped FeOOH (Na‐FeOOH) and Cl‐doped FeOOH (Cl‐FeOOH) acting as the cathode and anode, respectively, was rationally constructed to realize synergistically pseudocapacitive ion removal for effective desalination application. The Na‐ and Cl‐doped FeOOH materials were fabricated via a facile one‐step hydrothermal growth route, and in contrast to the *α*‐phase features of Na‐FeOOH and FeOOH, Cl‐FeOOH yielded a *β*‐phase property with a characteristic tunnel‐type structure (**Figure** **1**). Interestingly, the resultant FeOOH hybrids demonstrated a similar specific microstructure including enriched porosity, a highly open network, and hierarchical pore alignment, which all facilitated the efficient ion transfer towards the framework. Benefitting from the synergistic effects of specific structural features, increased electrochemical conductivity, and synergistically pseudocapacitive removal capability, the as‐assembled asymmetric Na‐FeOOH//Cl‐FeOOH cell manifested the best CDI capacity of up to 35.12 mg g^−1^ in a 500 mg L^−1^ NaCl solution and outstanding long‐term stability. To gain a better understanding of the remarkable CDI behavior of the hybrid cell, X‐ray diffraction (XRD) and ex‐situ X‐ray photoelectron spectroscopic (XPS) analysis were performed to further clarify the pseudocapacitive synergy removal mechanism.

## 2 Results and Discussion

**Figure 2:**
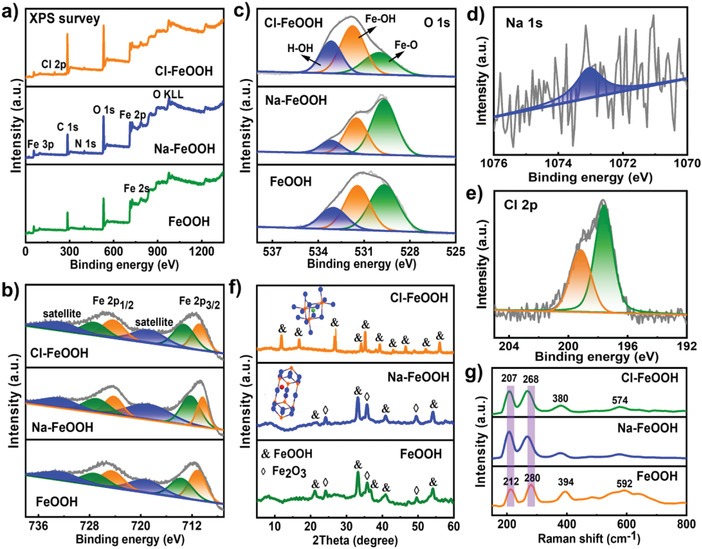
a) Full‐range and high‐resolution XPS spectra of b) Fe 2p and c) O 1s of as‐fabricated samples (top: Cl‐FeOOH; middle: Na‐FeOOH; bottom: bare FeOOH). High‐resolution XPS spectra of d) Na 1s and e) Cl 2p for Na‐FeOOH and Cl‐FeOOH. f) XRD patterns (insets were crystal structure diagrams by the Diamond software for Na‐FeOOH and Cl‐FeOOH) and g) Raman spectra of three as‐fabricated samples.

An ingenious and controllable one‐step hydrothermal strategy was developed to synthesize Na‐ and Cl‐FeOOH hybrids with NaOH and FeCl_3_ as doping precursors, respectively, as illustrated in Figure 1. An efficient transformation reaction between Fe^3+^ and OH^−^, mainly from urea hydrolysis, occurred under hydrothermal conditions in the presence of a PVP‐based structure‐directing surfactant. The surface elemental composition and atomic valence state of the as‐prepared FeOOH‐based hybrids were analyzed using X‐ray photoelectron spectroscopy (XPS). In addition to the inevitable C and N contamination that occurred upon air exposure (Figure S1, Supporting Information), the full‐range XPS spectra of bare FeOOH revealed the co‐existence of elements such as Fe and O without other impurities (**Figure** **2**a). First, the high‐resolution Fe 2p spectrum (Figure 2b) displayed pronounced binding energy peaks at 713.6/727.2 eV for Fe 2p_3/2_/Fe 2p_1/2_ with a separation spine‐orbit energy of ≈13.6 eV, which was in good agreement with the Fe^3+^ structure. At the same time, doublet peaks at 711.1/724.5 eV corresponded to the Fe 2p_3/2_/Fe 2p_1/2_ of the Fe^2+^ phases. Further, the high‐resolution O 1s XPS spectra (Figure 2c) showed three peaks at binding energies of 529.6, 531.4, and 533.0 eV, which can be ascribed to Fe─O, Fe─OH, and H─OH bonds, respectively.^[^ ^28^, ^51^ ^]^ These XPS observations indicated the successful construction of the FeOOH structure via a facile hydrothermal reaction. Second, after the NaOH dopant was added to the precursor solution, the resultant sample demonstrated a similar full‐range XPS survey without the presence of Na because of its relatively low mass loading. In particular, the co‐existence of two doublet peaks at 712.2/726.8 and 710.3/724.2 eV for Fe 2p_3/2_/Fe 2p_1/2_ and three peaks at 529.7, 531.5, and 533.2 eV in the high‐resolution Fe 2p and O 1s XPS spectra, respectively, both suggested a well‐maintained FeOOH structure even after the incorporation of the Na dopant. Moreover, compared to bare FeOOH, the high‐resolution Fe 2p spectrum of Na‐FeOOH showed a negative shift resulting from the strong charge‐transfer interaction between the FeOOH host and Na guest, indicating the effective insertion of Na ions into the FeOOH structure. Notably, the evident appearance of a relatively intense peak at 1073 eV in the high‐resolution Na 1s spectrum (Figure 2d) of Na‐FeOOH can be attributed to the Na phase,^[^ ^52^ ^]^ further suggesting its successful doping. The relative Na loading content was estimated to be 0.28 wt%, agreeing well with the value (0.24 wt%) determined by the inductively coupled plasma optical emission spectrometry (ICP‐OES) test (Table S1, Supporting Information). Third, after the replacement of Fe(NO_3_)_3_ with the FeCl_3_ precursor during the hydrothermal process accompanied by the simultaneous absence of the NaOH dopant, a novel FeOOH with an effective Cl ion dopant was able to be produced. Apart from the similar characteristic high‐resolution Fe 2p and O 1s spectra corresponding to the FeOOH phase, the apparent Cl element was clearly detected in the full‐range and high‐resolution XPS spectra of Cl‐FeOOH. Additionally, the Cl 2p peaks (Figure 2e) could be deconvoluted into two pronounced bands at 197.6 and 199.1 eV for Cl 2p_3/2_ and Cl 2p_1/2_, respectively, which also demonstrated that the Cl ions can be effectively inserted into the unique tunnel‐type structure of FeOOH via a robust one‐step hydrothermal method, and the relative Cl doping content was calculated to be 4.34 wt% via XPS analysis (Table S1, Supporting Information).^[^ ^31^ ^]^ In brief, it can be deduced that Na‐ and Cl‐doped FeOOH can be simply fabricated via controllable doping engineering.

X‐ray diffraction (XRD) measurements were performed to investigate the crystal structures of the as‐prepared samples. As shown in Figure 2f and Figure S2, Supporting Information, the diffraction peaks located at 21.2°, 33.1°, 34.6°, 36.7°, 41.1°, and 53.1° belonged to the characteristic planes of the (110), (130), (021), (111), (140), and (221) planes (JCPDS No. 81–0462), respectively, which corresponded well to the orthogonal *α*‐FeOOH structure. In addition, the three typical peaks at 24.2°, 35.6°, and 49.5° can be ascribed to the Fe_2_O_3_ phase (JCPDS No. 33–0664). The XRD results indicated that the bare sample was composed of the FeOOH/Fe_2_O_3_ hybrid, and the resultant FeOOH showed an *α*‐phase open structure, which benefited from the efficient ion transfer pathway and excellent structural stability. ^[^ ^35^, ^53^ ^]^ Importantly, compared to bare FeOOH, no new peaks appeared for the Na‐FeOOH sample, suggesting that the doped Na ions did not affect the crystal structure deformation of FeOOH, and the unique *α*‐phase structure was well maintained. Nevertheless, the peak intensity of Fe_2_O_3_ increased to a certain, which may have resulted from the slight surface oxidation of FeOOH in the alkaline environment after the addition of NaOH.^[^ ^41^ ^]^ Notably, after utilizing FeCl_3_ as a substitute for FeNO_3_, a distinctive FeOOH hybrid structure was produced. Several characteristic peaks centered at 11.8°, 16.8°, 26.8°, 33.9°, 35.3°, 39.1°, 46.6°, 52.2°, and 56.0° were attributed to the (110), (200), (310), (400), (211), (301), (411), (600), and (521) planes, respectively, of the *β*‐FeOOH phase (JCPDS No. 34–1266) with a tetragonal structure, which was significantly different from those of the bare FeOOH and Na‐FeOOH samples.^[^ ^54^, ^55^ ^]^ Thus, the XRD observations further showed the successful fabrication of FeOOH, in accordance with the XPS results, and two different FeOOH phases, *α* and *β*, could be obtained by controlling the synthetic methodology with the varying Fe‐based precursors. Furthermore, Raman spectroscopy, shown in Figure 2g, revealed the chemical components of the three samples. Similar Raman spectra were obtained for bare FeOOH, Na‐FeOOH, and Cl‐FeOOH. The pronounced Raman bands located at 212, 280, 394, and 592 cm^−1^ were indexed to the dissymmetric stretching vibration between the metal and hydroxide groups, consistent with the typical FeOOH crystalline feature.^[^ ^56^ ^]^ Notably, the corresponding typical peaks of Na‐FeOOH and Cl‐FeOOH demonstrated a slight blue shift originating from the evident ion doping of the FeOOH microstructure.

**Figure 3:**
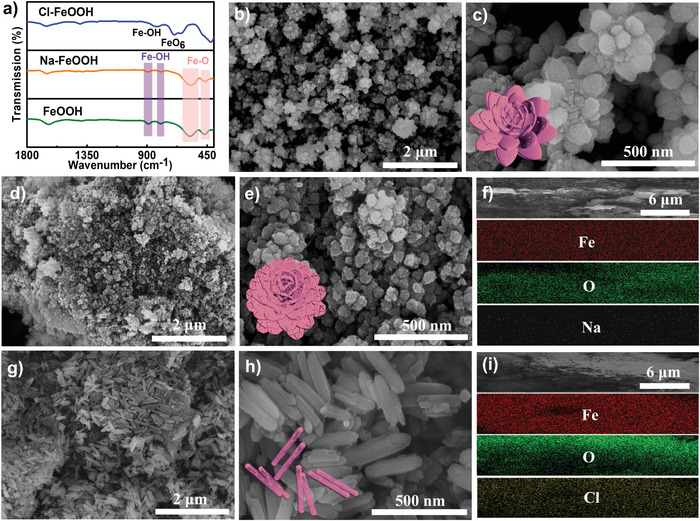
a) FT‐IR spectra of three as‐fabricated samples. SEM images of b,c) bare FeOOH, d,e) Na‐FeOOH and g,h) Cl‐FeOOH. Corresponding elemental mapping images of f) Na‐FeOOH and i) Cl‐FeOOH.

Additionally, characteristic surface functional groups and molecular structures were monitored via Fourier‐transform infra‐red (FT‐IR) spectroscopy. As depicted in **Figure** **3**a, similar to those of the FeOOH, two sharp absorption peaks at 889 and 798 cm^−1^, corresponding to the Fe─OH bending vibrations, were detected for the Na‐FeOOH hybrid. In addition, the two intense bands at 579 and 469 cm^−1^ corresponded to the Fe─O stretching and bending vibrations, respectively, which were assigned to the unique vibration modes of the *α*‐FeOOH phase.^[^ ^53^ ^]^ Interestingly, in the FT‐IR spectrum for Cl‐FeOOH, apart from the typical absorption peaks of Fe─O at 425 cm^−1^ and Fe‐OH at 840 cm^−1^, the new peak at 682 cm^−1^ was indexed to the FeO_6_ coordination octahedron unit cell, corresponding to the *β*‐FeOOH phase.^[^ ^57^ ^]^ By coupling XPS, ICP‐OES, XRD, and Raman spectroscopy with FT‐IR observations, it can be concluded that Na‐FeOOH and Cl‐FeOOH hybrids with *α* and *β* phases, respectively, were successfully prepared via robust hydrothermal growth‐assisted controllable doping engineering.

**Figure 4:**
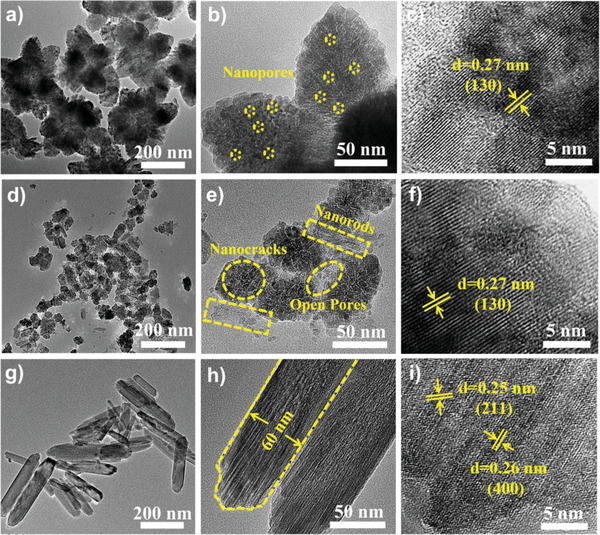
TEM images of a–c) bare FeOOH, d–f) Na‐FeOOH, and g–i) Cl‐FeOOH under different magnifications.

The morphologies and microstructures of the samples were revealed using the transmission electron microscopy (TEM) and scanning electron microscopy (SEM). As illustrated in Figure 3b,c and Figure S3a,b, Supporting Information showed that, in the presence of the PVP surfactant, bare FeOOH exhibited a well‐arranged and homogenous nanoflower‐like morphology with a mean diameter of ≈415 nm, which was assembled by interconnected nanorods, and enriched macropores were also produced among the adjacent flowers. Notably, after the Na‐ion doping of the FeOOH network, the resultant Na‐FeOOH demonstrated an evidently different microstructure (Figure 3d,e and Figure S3c,d, Supporting Information). First, it exhibited a quasi‐flower‐like feature with an evident morphology deformation from bare FeOOH. Second, Na‐FeOOH exhibited a much smaller average lateral size of ≈70 nm (Figure 3e). Therefore, Na‐ion doping reached the morphology engineering and microstructure transformation of the bare FeOOH. Nevertheless, the characteristic porous framework was well maintained, strengthening an efficient ion diffusion/transfer pathway across the electrode network during the CDI process. Interestingly, when the Cl‐based doping precursor was utilized during the hydrothermal reaction, Cl‐FeOOH manifested a completely different structural feature (Figure 3g,h and Figure S3e,f, Supporting Information), and the resultant hybrid obtained a nanorod morphology with a random arrangement. Each rod exhibited a mean length of ≈250 nm and a width of about 60 nm (Figure 3g,h). In addition, EDS mapping suggested the even distribution of Fe, O, and Na elements without other impurities in Na‐FeOOH (Figure 3f), while the Fe, O, and Cl elements were evenly distributed in the Cl‐FeOOH hybrid (Figure 3i), further revealing that the successful doping of guest ions such as Na^+^ and Cl^−^ into the bare FeOOH framework, was well matched with the XPS, ICP‐OES, and FT‐IR results. Moreover, the TEM images of bare FeOOH (**Figure** **4**a,b) displayed a uniform flower‐based structure constructed with well‐interconnected nanorods (≈12 nm in width and 140 nm in length). Additionally, enriched nanopores of about 3 nm clearly existed throughout the nanosheets, and the characteristic lattice fringe of 0.27 nm corresponded well to the (211) plane of FeOOH (Figure 4c). In contrast, the Na‐FeOOH hybrid exhibited quasi‐flower‐like structures with significantly decreased lateral sizes, while abundant nanocracks and open pores existed across the framework (Figure 4d,e). However, an interplanar distance of about 0.27 nm was also seen in Na‐FeOOH (Figure 4f), indicating the well‐preserved crystalline structure of FeOOH. Finally, as observed in Figure 4g,h, Cl‐FeOOH reflected a well‐refined nanorod feature with a length of ≈250 nm and a width of 60 nm, which agreed well with the SEM observations. Also, the HR‐TEM image (Figure 4i) showed interplanar distances of ≈0.25 and 0.26 nm, which were indexed to the (211) and (400) diffraction planes, respectively, of the anatase Cl‐FeOOH nanorods.^[^ ^36^, ^58^ ^]^ As revealed by the N_2_ adsorption‐desorption isotherms (Figures S4, S5a,c, Supporting Information and Table S1, Supporting Information), bare FeOOH, Na‐FeOOH, and Cl‐FeOOH further confirmed the co‐existence of mesopore and macropore features, ensuring a robust ion transfer pathway across the electrode framework.

**Figure 5:**
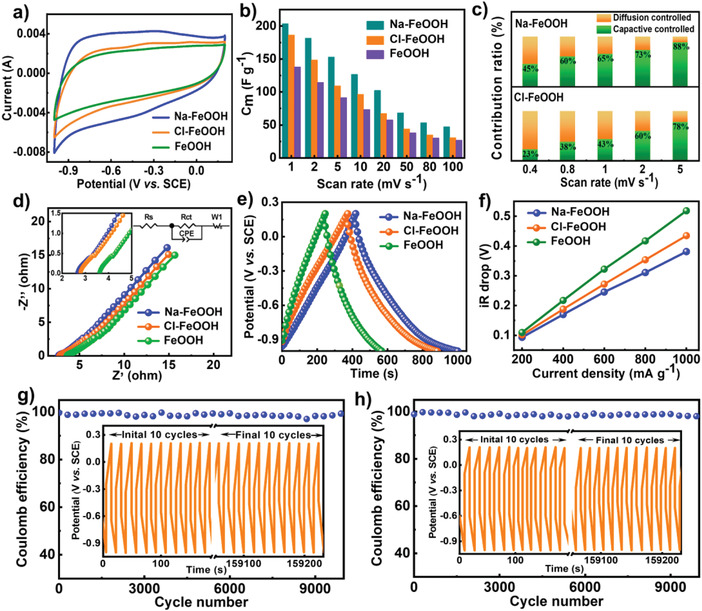
Electrochemical performance tests of three as‐fabricated electrodes in 1 m NaCl solution: a) comparative CV curves at scan rate of 5 mV s^−1^; b) calculated *C*_m_ versus scan rates; c) capacity contributions of Na‐FeOOH and Cl‐FeOOH at different scan rates; d) Nyquist impedance curves (insets were fitted circuit diagram by the ZView software and high‐frequency region curve); e) GCD plots at current density of 200 mA g^−1^; f) *iR* drops versus current loads; resultant Coulomb efficiency versus cycle number plots of g) Na‐FeOOH and h) Cl‐FeOOH (inset was the corresponding GCD cycling plot at current density of 3000 mA g^−1^ for each electrode).

To assess the electrochemical capacity performance of the three electrodes, cyclic voltammetry (CV) measurements were first carried out within a potential window of −1–0.2 V (vs the saturated calomel electrode, (SCE)) in a traditional three‐electrode system. As illustrated in **Figure** **5**a, compared with that of the bare FeOOH, the resultant closed CV curve areas of the Na‐FeOOH and Cl‐FeOOH hybrid electrodes both increased significantly, reflecting the enhanced deionization capacity. As quantitatively determined, specific capacitances of the Na‐FeOOH and Cl‐FeOOH electrodes reached 153.0 and 109.3 F g^−1^, respectively, which exceeded that of the FeOOH electrode (91.3 F g^−1^) remarkably at scan rate of 5 mV s^−1^. Interestingly, the Na‐FeOOH electrode reached the maximum capacity under similar test conditions, which resulted from the superior electrochemical properties promoted by high‐conductivity Na doping and the specific microstructure. Thus, the deionization behavior of FeOOH can be optimized via rational surface doping engineering, thus ensuring FeOOH‐based composites as promising CDI electrode alternatives for highly efficient desalination applications. In addition, the rate capability was determined by CV measurements from 1 to 100 mV s^−1^ (Figure 5b and Figure S6a–c, Supporting Information), and the calculated specific capacitance decreased with an increasing rate. Capacity degeneration was associated with insufficient ion accessibility in the internal pore network and the enlarged ion diffusion resistance at a higher operation rate. Notably, the Na‐FeOOH hybrid electrode demonstrated the maximum deionization capacity at all scan rates.

Importantly, the co‐appearance of oxidation/reduction peaks in the CV curves implied a reversible faradaic reaction corresponding to the ion intercalation/deintercalation towards the hybrid electrodes.^[^ ^48^ ^]^ To further explore the charge transfer mechanism during the desalination process, CV curves were recorded at various sweep rates from 0.4 to 5 mV s^−1^, and the pseudocapacitance/diffusion‐controlled capacity contributions were calculated accordingly (Figure 5c, Figures S7a,b, S8a,b, Supporting Information). A pair of redox peaks was clearly detected at any chosen scan rate, further suggesting electrochemical faradaic reactions between the Fe^3+^ and Fe^2+^ phases of FeOOH (e.g., Fe^3+^ + e^−^ ⇌ Fe^2+^) promoted by the ion intercalation/deintercalation process. The calculated pseudocapacitive contribution showed an evident increase with an increasing running rate, and the maximum values reached ≈88% and 78% at scan rate of 5 mV s^−1^ for the Na‐FeOOH and Cl‐FeOOH electrodes, respectively. Therefore, it can be deduced that the pseudocapacitance behavior determined the deionization performance of FeOOH electrodes.

The electrochemical conductivity, a crucial factor in CDI performance, was verified by electrochemical impedance spectroscopy (EIS) with a three‐electrode system similar to that used in the CV tests. As exhibited in Figure 5d, the resultant Nyquist plots demonstrated a quasi‐semicircle in the high‐frequency region and a straight segment in the low‐frequency region, which mainly corresponded to the charge‐transfer resistance (*R_ct_*) and Warburg diffusion impedance (*W*), respectively. Both the Na‐FeOOH and Cl‐FeOOH hybrid electrodes manifested pronouncedly smaller intersection values with the X‐coordinates in comparison to that of the bare FeOOH, indicating their alleviated charge‐transfer impedance. Furthermore, a simulated equivalent circuit (inset of Figure 5d) was also provided, in which *R_s_* and CPE represented the inherent solution resistance and constant phase element part, respectively. The fitted *R_ct_* values were calculated to be 5.18, 5.22, and 7.47 Ω for Na‐FeOOH, Cl‐FeOOH, and FeOOH electrodes, respectively, and the steeper slope lines in the low‐frequency region indicated the excellent capacitive characteristic (Figure S9, Supporting Information). Notably, compared to that of bare FeOOH (3.15 × 10^−15^ cm^2^ s^−1^), the ion diffusion coefficients were determined to be approximately 3.30 × 10^−15^ and 3.21 × 10^−15^ cm^2^ s^−1^ for the Na‐FeOOH and Cl‐FeOOH, respectively, revealing the superiority of the hybrid electrodes. Thus, based on the above observations, the doped FeOOH electrodes showed improved electrochemical conductivity, and the Na‐FeOOH electrode exhibited the best conductive property. The outstanding conductivity performance can be ascribed to the following aspects: first, the highly open pore network, hierarchical pore arrangement, and numerous nanocracks, as displayed by the SEM, TEM, and N_2_ adsorption‐desorption isotherms, benefited the deceased ion/electron transfer and diffusion resistances throughout the electrode as well as the bulk solution; second, compared to Cl‐FeOOH and bare FeOOH, the highly conductive Na incorporation can further boost the electrochemical conductivity. Thus, the synergistic effects of the specific microstructure and Na doping endowed the Na‐FeOOH hybrid electrode with the highest conductivity.

In addition, to further clarify the pronounced superiority of FeOOH hybrid electrodes in terms of electrochemical capacity and conductivity, galvanostatic charge–discharge (GCD) measurements at varying current loads were conducted in detail. As depicted in Figure 5e and Figure S10, Supporting Information, the resultant GCD curves exhibited relatively symmetric styles with quasi‐triangular shapes, indicating the outstanding capacitive behavior of the three electrodes. More importantly, the discharge time for the Na‐FeOOH and Cl‐FeOOH electrodes was prolonged in comparison to that of bare FeOOH, and Na‐FeOOH showed the maximum value, verifying the highest electrochemical capacitance, which was consistent with the CV results. Subsequently, the *iR* drop assigned to the inherent electrode resistance was detected (Figure 5f and Figure S10, Supporting Information). The *iR* drop versus current load curve displayed a decreased electrical resistance associated with the improved electrochemical conductivity of the two hybrid electrodes, and Na‐FeOOH possessed the minimum value at any operation load, corresponding well with the EIS observations. The cycling stability was another crucial parameter for high‐performance CDI electrodes. As shown in Figure 5g,h, the consecutive GCD cycling curves of both Na‐FeOOH and Cl‐FeOOH electrodes maintained their initial shape well even after 10 000 cycles at current load of 3000 mA g^−1^, and the mean Coulomb efficiency was ≈98%, certificating the outstanding long‐term durability of the hybrid electrodes. Briefly, the coupling of the CV, EIS, and GCD results demonstrated the superior electrochemical property (e.g., capacity, conductivity, and stability) of the as‐constructed hybrid electrodes, which ensured their great potential for efficient CDI applications.

**Figure 6:**
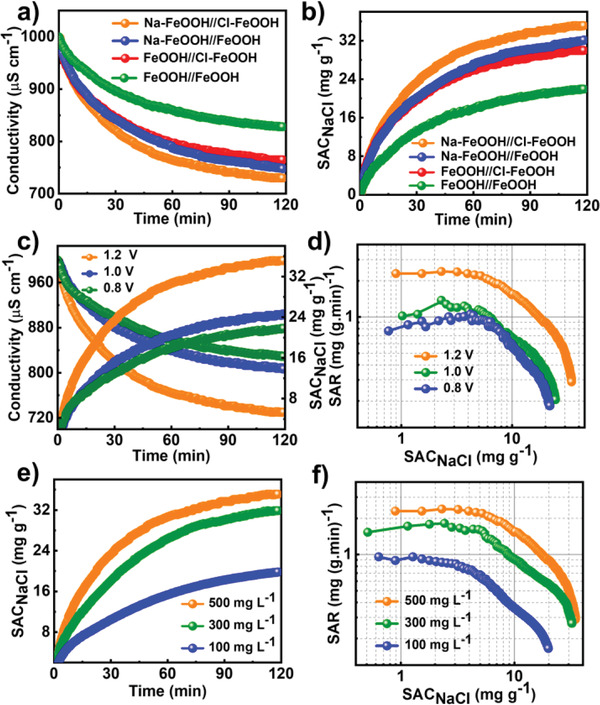
a) Dynamic solution conductivity versus running time and b) corresponding SAC_NaCl_ versus running time plots of different CDI cells in NaCl aqueous solution of 500 mg L^−1^ with an external voltage of 1.2 V. c) Dynamic solution conductivity/SAC_NaCl_ versus running time and d) Ragone plots of Na‐FeOOH//Cl‐FeOOH cell with initial concentration of 500 mg L^−1^ using different voltages. e) SAC_NaCl_ versus running time and f) Ragone plots of Na‐FeOOH//Cl‐FeOOH cell in NaCl aqueous solution with different concentrations using an applied voltage of 1.2 V.

The CDI performance evaluation in a simulated NaCl solution was an effective avenue for investigating the promising potential of as‐fabricated FeOOH‐based hybrid electrodes for desalination applications. The deionization behavior was measured in an asymmetric CDI cell (Na‐FeOOH//Cl‐FeOOH) consisting of Na‐FeOOH and Cl‐FeOOH electrodes working as the cathode and anode, respectively. Here, the Na^+^ in the effluent was transferred and further intercalated into the 3D hierarchical quasi‐flower‐like Na‐FeOOH microstructure accompanied by simultaneous Cl^−^ intercalation towards the Cl‐FeOOH electrode upon the driven voltage, and an efficient desalination process was achieved. To highlight the deionization superiority of the Na‐FeOOH//Cl‐FeOOH cell for CDI water treatment, Na‐FeOOH//FeOOH, FeOOH//Cl‐FeOOH, and bare FeOOH//FeOOH cells were also assembled reasonably for comparison under similar operating conditions. First, the CDI test was performed in a NaCl solution of 500 mg L^−1^, and the resultant salt adsorption capacity (SAC_NaCl_) was quantitatively determined by the linear relationship between the outlet solution conductivity and concentration. As illustrated in **Figure** **6**a, the dynamic conductivity declined remarkably under an applied forward voltage of 1.2 V, demonstrating the effective capture of Na and Cl ions in the Na‐FeOOH//Cl‐FeOOH cell. With prolonged running time, the conductivity decline rate was alleviated to some extent, and it reached electrochemical equilibrium after ≈120 min. More importantly, the calculated SAC_NaCl_ of the Na‐FeOOH//Cl‐FeOOH cell reached up to 35.12 mg g^−1^, markedly exceeding that of the Na‐FeOOH//FeOOH (32.13 mg g^−1^), FeOOH//Cl‐FeOOH (30.22 mg g^−1^), and bare FeOOH//FeOOH cells (22.06 mg g^−1^), demonstrating its superior ion capture performance (Figure 6b). Further, the charge efficiency and energy consumption of single electrode were determined to be 0.88 and 0.62 Wh g^−1^, respectively (Figure S11, Supporting Information). Based on these results, it can be deduced that the rational doping of Na and Cl guests can effectively boost the CDI property of bare FeOOH electrode. Moreover, the effects of the working parameters comprising the working voltage and initial effluent concentration on the CDI performance of Na‐FeOOH//Cl‐FeOOH cells were further verified. As clearly observed in Figure 6c,d, the SAC_NaCl_ values manifested a pronounced improvement with increasing voltage, which was ascribed to the enhanced ion intercalation towards the electrodes driven by the enlarged electrostatic force. Furthermore, at a higher voltage, the Ragone plots showed an evident shift to the upper‐right region, also suggesting improved CDI properties (e.g., capacity and removal rate). Additionally, a superior SAC_NaCl_ and salt adsorption rate (SAR_NaCl_) can be realized at higher solution concentrations. A SAC_NaCl_ value of 19.86 mg g^−1^ was obtained for 100 mg L^−1^, and the value reached a maximum value of 35.12 mg g^−1^ for 500 mg L^−1^ (Figure 6e,f and Figure S12a,b, Supporting Information). Thus, the cell operating parameters (e.g., working voltage and effluent concentration) had significant effects on CDI performance. Notably, the as‐constructed Na‐FeOOH//Cl‐FeOOH cell exhibited superior removal capacity compared to the reported carbon‐ and TMC‐based CDI electrodes (Table S2, Supporting Information).

**Figure 7:**
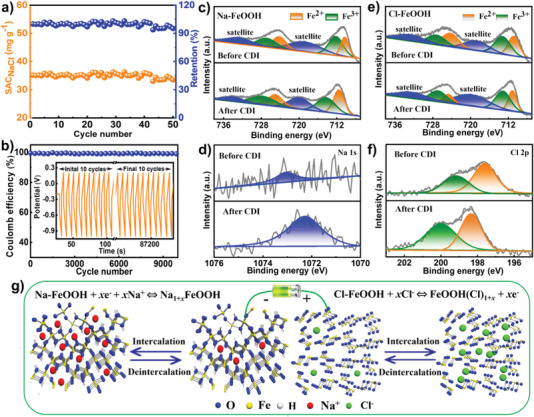
Cycling performance tests of Na‐FeOOH//Cl‐FeOOH cell: a) dynamic SAC_NaCl_/capacity retention versus cycle number in NaCl aqueous solution with initial concentration of 500 mg L^−1^ using an applied voltage of 1.2 V; b) Coulomb efficiency versus cycle number in 1 m NaCl aqueous solution (inset was the corresponding GCD cycling plot at current density of 2000 mA g^−1^). High‐resolution c) Fe 2p and d) Na 1s spectra of Na‐FeOOH electrode before and after Na^+^ intercalation. High‐resolution e) Fe 2p and f) Cl 2p spectra of Cl‐FeOOH electrode before and after Cl^−^ intercalation. g) Schematic illustrations of the contribution of synergetic pseudocapacitive removal from asymmetric Na‐FeOOH//Cl‐FeOOH CDI cell.

It was well known, the regeneration performance, also known as the cycling stability, was another important evaluation factor for efficient CDI electrodes. Consecutive CDI intercalation/deintercalation tests of 50 cycles were carried out in a 500 mg L^−1^ NaCl solution for the Na‐FeOOH//Cl‐FeOOH cell. As shown in **Figure** **7**a, an outstanding SAC_NaCl_ retention of ≈100% was observed, and the solution conductivity demonstrated no pronounced decline and a relatively stable pH value throughout the prolonged running time (Figures S13, S14, Supporting Information). Consecutive GCD cycling evaluations were performed to gain further insight into the excellent durability of the as‐constructed asymmetric CDI configuration. As expected, GCD cycling plots retained similar shapes without discharge time degradation, and the corresponding Coulomb efficiency reserved ≈100% without an evident attenuation even after 10 000 cycles (Figure 7b). These results revealed that the Na‐FeOOH//Cl‐FeOOH cell possessed robust durability, which may be owing to the highly stable Na‐FeOOH and Cl‐FeOOH electrodes, as illustrated by their corresponding GCD tests (Figure 5g,h). Additionally, XRD patterns (Figure S15, Supporting Information) and SEM images (Figure S16, Supporting Information) after the CDI process demonstrated well‐maintained crystal structure and surface morphology of the hybrid framework, respectively, further illustrating the outstanding structural stability of hybrid electrodes, agreeing well with the GCD and CDI cycling tests. Meanwhile, no evident new diffraction peaks were detected which suggested the free Na/Cl ion accessibility of the open FeOOH architecture. In brief, compared with the bare cell (e.g., FeOOH//FeOOH), all doped asymmetric cells (e.g., Na‐FeOOH//Cl‐FeOOH, Na‐FeOOH//FeOOH, and FeOOH//Cl‐FeOOH) exhibited enhanced desalination behavior, and the best performance was observed in the Na‐FeOOH//Cl‐FeOOH cell with the Na‐FeOOH and Cl‐FeOOH electrodes acting as the cathode and anode, respectively, for superior Na and Cl ion capture. The superior CDI performance (e.g., desalination capacity and stability) can be attributed to three aspects: first, the specific structural features (e.g., enriched porosity, 3D hierarchical pore alignment, and highly open crystalline framework) facilitated efficient ion/electron diffusion and transfer across the hybrid electrode framework; second, the rational doping engineering of FeOOH with both Na and Cl ions endowed the electrode with enhanced electrochemical conductivity; third, the doped Na/Cl ions existing in the open structure of FeOOH may provide efficient ion exchange between the electrode and the bulk solution to further optimize the ion transfer pathway, and effectively promoted the faradaic reaction of FeOOH during the CDI process.

To reveal the working mechanism for the boosting CDI performance of Na‐FeOOH//Cl‐FeOOH cell, detailed XPS tests were performed. The XPS survey spectra of the Na‐FeOOH and Cl‐FeOOH hybrid electrodes before and after CDI process were recorded, as illustrated in Figure 7c–f. Compared to the fresh one, the high‐resolution Fe 2p spectrum (Figure 7c) for the Na‐FeOOH electrode manifested an increasing Fe^2+^ content accompanied by a remarkably decreased content of Fe^3+^ after the efficient intercalation of the Na^+^ guest. In addition, the typical peak assigned to Na showed an evident negative shift, and the peak intensity realized a significant improvement (Figure 7d), demonstrating the successful embedding of Na^+^ into the Na‐FeOOH electrode. Therefore, it can be deduced that the Na‐FeOOH electrode clarified the reversible faradaic reaction conversion of the Fe^3+^/Fe^2+^ pair (Na‐FeOOH + *x*e^−^ + *x*Na^+^ ⇔ Na_1+_ *_x_* FeOOH) for efficient Na‐ion removal/release. Notably, in contrast to that of Na‐FeOOH, the weakened peak intensity ascribed to the Fe^2+^ phase was determined by the high‐resolution Fe 2p spectrum (Figure 7e) of the Cl‐FeOOH hybrid electrode, whereas the Fe^3+^ phase showed an enhanced peak intensity after Cl^−^ intercalation. Additionally, the high‐resolution Cl 2p spectra of Cl‐FeOOH before and after desalination were obtained. Two pronounced deconvoluted peaks at 198.3 and 199.7 eV corresponded to Cl 2p_3/2_ and Cl 2p_1/2_, respectively, and the resultant binding energies displayed a relatively positive shift in comparison to that of the fresh Cl‐FeOOH electrode, demonstrating the successful Cl ion intercalation towards the hybrid network. Therefore, an evident electrochemical reaction (Cl‐FeOOH + *x*Cl^−^ ⇔ FeOOH(Cl)_1+_ *_x_* + *x*e^−^) occurred for Cl^−^ capture on the Cl‐FeOOH electrode. Thus, it was inferred that the synergy of the co‐existing pseudocapacitance contribution from the Na‐FeOOH cathode and Cl‐FeOOH anode led to the superior CDI performance of the asymmetric Na‐FeOOH//Cl‐FeOOH cell (Figure 7g).

## 3 Conclusion

In summary, we report the successful fabrication of two novel FeOOH hybrid heterostructures (Na‐FeOOH and Cl‐FeOOH) via facile hydrothermal growth‐based controllable precursor doping engineering. As robust CDI electrodes, the asymmetric Na‐FeOOH//Cl‐FeOOH cell exhibited a remarkable SAC_NaCl_ of 35.12 mg g^−1^ in a 500 mg L^−1^ NaCl solution and outstanding long‐term durability. The excellent CDI performance of the Na‐FeOOH//Cl‐FeOOH cell can be attributed to unique structural features (e.g., hierarchical pore texture, highly open porosity, and freely accessible crystalline microstructure), superior electrochemical conductivity, and advanced optimized ion diffusion properties resulting from the enhanced Na/Cl ion exchange between the hybrid electrode and the bulk solution. In addition, the synergy of the pseudocapacitive contributions from the Na‐FeOOH cathode and Cl‐FeOOH anode was demonstrated via XRD and XPS analyzes. Our work may provide an efficient methodology for the design and development of novel FeOOH hybrids for the boosting CDI application.

## 4 Experimental Section

### Materials and Chemicals

the ferric nitrate nonahydrate (Fe(NO_3_)_3_·9H_2_O, 98.00%), ferric chloride (FeCl_3_, 98.00%), urea (NH_2_CONH_2_, ≥ 99.00%), sodium hydroxide (NaOH) and polyvinylpyrrolidone (PVP) were supplied by Shanghai Titan Scientific Co., and utilized directly as received without further purification. Deionized water (H_2_O) was used throughout the experiments.

### Synthesis of Na‐FeOOH Hybrid Nanoflowers

First, the Na‐FeOOH quasi‐flower‐like powder was prepared using a facile one‐step hydrothermal strategy as illustrated in Figure 1. Quantities of 832 mg of Fe(NO_3_)_3_·9H_2_O, 12 mg of NH_2_CONH_2_, and 75 mg of PVP were dissolved in 20 mL of H_2_O via ultrasonic treatment. Then, 26.5 mg of NaOH was slowly added to the mixture, and the suspension was further transferred into a Teflon reactor (50 mL) and maintained at 100 °C for 12 h. After cooling to room temperature, the light‐yellow precipitate was completely washed with H_2_O and ethanol several times, and the final hybrid powder, denoted as Na‐FeOOH, was dried at 40 °C for 12 h under vacuum. For comparison, bare FeOOH powder was also fabricated using a hydrothermal process similar to that of the Na‐FeOOH hybrid in the absence of NaOH dopant.

### Synthesis of Cl‐FeOOH Nanorods

First, 343 mg of FeCl_3_, 12 mg of NH_2_CONH_2_, and 75 mg of PVP were added to 20 mL of H_2_O under magnetic stirring. Afterwards, the mixture solution was transferred to a Teflon reactor (50 mL) and maintained at 100 °C for 12 h. Then, the powder was washed adequately with H_2_O and ethanol. Finally, the yellow powder was dried at 40 °C for 12 h under vacuum to obtain the Cl‐doped FeOOH hybrid (e.g., Cl‐FeOOH).

### Structural Characterizations and Electrochemical Performance Measurements

The morphologies and microstructures of the Na‐FeOOH, Cl‐FeOOH, and FeOOH materials were characterized via X‐ray photoelectron spectroscopy (XPS), inductively coupled plasma optical emission spectrometry (ICP‐OES), X‐ray diffraction (XRD), Raman spectroscopy, Fourier‐transform infra‐red spectroscopy (FT‐IR), scanning electron microscopy (SEM), high‐resolution transmission electron microscopy (HR‐TEM), and N_2_ adsorption‐desorption tests. The electrochemical properties of the corresponding electrodes were evaluated by cyclic voltammetry (CV), electrochemical impedance spectroscopy (EIS), and galvanostatic charge/discharge (GCD) using an electrochemical workstation and an automatic LAND battery cell, respectively. All electrochemical tests with a standard three‐electrode cell were performed at room temperature in 1 m NaCl solution. More details on the structural characterizations and electrochemical measurements were provided in Supporting Information.

### Desalination Behavior Measurements

The FeOOH, Na‐FeOOH, and Cl‐FeOOH CDI electrodes were constructed via traditional powder electrode processing at a size of 8 cm × 11 cm × 0.1 cm.^[^ ^21^ ^]^ A self‐made CDI cell was assembled to evaluate desalination performance with Cl‐FeOOH and Na‐FeOOH electrodes acting as the anode and cathode, respectively, and the resultant asymmetric Na‐FeOOH//Cl‐FeOOH cell was constructed. Na‐FeOOH//FeOOH, FeOOH//Cl‐FeOOH, and bare FeOOH//FeOOH cells were also fabricated for comparison. In the CDI operation, working parameters of solution volume, running time, and flow rate were 50 mL, 120 min, and 60 mL min^−1^, respectively. The dynamic outlet solution concentration was determined using the recorded real‐time conductivity. To further optimize the CDI property of the asymmetric Na‐FeOOH//Cl‐FeOOH cell, the effects of factors such as solution concentration (e.g, 100, 300, and 500 mg L^−1^) and driven voltage (e.g., 0.8, 1.0, and 1.2 V) were also evaluated.

The salt adsorption capacity (SAC_NaCl_, mg g^−1^) and salt adsorption rate (SAR_NaCl_, mg (g min)^−1^) were defined by Equations (1) and (2), respectively.

(1) $${\mathrm{SAC}}_{\mathrm{NaCl}}\;=\;\left(C_{0}-C\right)\;V\;/\;m$$

(2) $${\mathrm{SAR}}_{\mathrm{NaCl}}\;=\;\mathrm{SA}C_{\mathrm{NaCl}}\;/\;t$$

where *m* (g) represented the total active mass of the cathode and anode (e.g., 0.2 g), and *V* (L) depicted the rotational solution volume. *C_0_* and *C* (mg L^−1^) were the initial and final concentrations, respectively, and *t* (min) was the running time.

Capacity retention defined as the ratio of SAC_NaCl (_ *_n_*_)_ to SAC_NaCl (_ *_1_*_)_ was expressed by Equation (3).

(3) $$\mathrm{Retention}\;={\;\mathrm{SAC}}_{\mathrm{NaCl}(n)\;}\;/\;\mathrm{SA}C_{\mathrm{NaCl}(1)}$$

where SAC_NaCl (_ *_n_*_)_ and SAC_NaCl (_ *_1_*_)_ were the capacities at any CDI cycle and initial cycle, respectively, and *n* corresponded to the cycle number.

### Statistical Analysis

The output performance (e.g., current, time, voltage, resistance, frequency, solution conductivity) of CV, GCD, EIS, and conductivity meter were directly utilized from the original data collected by the aforementioned measurement techniques. On the basis of the recorded data, specific capacitance, capacity contribution, *iR* drop, Coulomb efficiency, diffusion coefficient, salt adsorption capacity, salt adsorption rate, capacity retention, charge efficiency, and energy consumption were calculated accordingly. Data analyzing/processing was operated by the Origin software.

## Conflict of Interest

The authors declare no conflict of interest.

## Supporting information

Supporting Information

## Data Availability

The data that support the findings of this study are available from the corresponding author upon reasonable request.
