# Supplementary material for: Efficient and Durable Sodium, Chloride‐doped Iron Oxide‐Hydroxide Nanohybrid‐Promoted Capacitive Deionization of Saline Water via Synergetic Pseudocapacitive Process
Source: Adv Sci (Weinh). 2022 Jul 11;9(25):2201678. doi: 10.1002/advs.202201678 (PMC9443451; doi:10.1002/advs.202201678)
Supplement: Supplementary file 1 — Supporting Information [file ADVS-9-2201678-s001.pdf]

ADVANCED SCIENCE
Open Access

Supporting Information

for *Adv. Sci.*, DOI 10.1002/advs.202201678

Efficient and Durable Sodium, Chloride-doped Iron Oxide-Hydroxide Nanohybrid-Promoted Capacitive Deionization of Saline Water via Synergetic Pseudocapacitive Process

Jingxuan Zhao, Bingyao Wu, Xinwei Huang, Yang Sun, Zhibo Zhao, Meidan Ye and Xiaoru Wen**

Supporting Information

Efficient and Durable Sodium, Chloride-doped Iron Oxide-Hydroxide Nanohybrid-Promoted Capacitive Deionization of Saline Water via Synergetic Pseudocapacitive Process

Jingxuan Zhao, Bingyao Wu, Xinwei Huang, Yang Sun, Zhibo Zhao, Meidan Ye, and Xiaoru Wen**

J. Zhao, B. Wu, X. Huang, Y. Sun, Z. Zhao, Prof. X. Wen
College of Chemistry and Chemical Engineering
Inner Mongolia University
Hohhot 010021, P. R. China
E-mail: xiaoru-wen@outlook.com
Prof. M. Ye, Z. Zhao
Research Institute for Biomimetics and Soft Matter
Fujian Provincial Key Laboratory for Soft Functional Materials Research
Department of Physics
College of Physical Science and Technology
Xiamen University
Xiamen 361005, P. R. China
Email: mdye@xmu.edu.cn

S1. Experiments

Material characterization: Elemental composition and surface state of the Cl-FeOOH, Na-FeOOH, and FeOOH samples were detailly studied by X-ray photoelectron spectroscopy (XPS), which was implemented on a VG Scientific ESCALAB Mark II spectrometer, and the spectra were calibrated with the C-C peak of 284.8 eV. To further characterize the phase composition, X-ray diffraction (XRD) test was carried out with a Rigaku D/MAX-RB X-ray diffractometer using the Cu Kα (40 kV, 20 mA) radiation and a secondary beam graphite monochromator. The surface chemical composition was identified using Fourier-transform infra-red spectroscopy (FT-IR, Bruker VERTEX70, Germany) operated at room temperature. Morphology and microstructure of FeOOH hybrid samples were observed via field emission scanning electron microscopy (SEM, Hitachi S-4800) and transmission electron microscopy (TEM, FEI Tecnai F20). Raman spectra were obtained on an optical microscope with the excitation of 514.5 nm line from an Ar^+ ion laser (Spectra Physics). Nitrogen adsorption-desorption isotherms were provided by ASAP 2020 Brunauer-Emmett-Teller (BET) analyzer, and specific surface area and pore volume properties were calculated by the BET method along with the estimated pore size distribution via Barrett-Joyner-Halenda (BJH) model.

Electrochemical performance measurements: Cyclic voltammetry (CV) plot was tested on the electrochemical workstation (CHI 660E) in a standard three-electrode system consisting of prepared electrode, graphite paper, and saturated calomel electrode (SCE) as the working, counter, and reference electrodes, respectively, and the specific capacitance (C_m, F g^{-1}) was calculated by Equation S1.

$$C_m = Q / mv \triangle V \quad \text{(S1)}$$

where m, $\triangle V$, and v indicated the active mass, chosen potential range, and running rate, respectively. The Q was the integrated CV curve area.

GCD measurements were conducted using an automatic LAND battery test instrument (Land CT2001A) to evaluate both capacitive property and cycling performance. Coulomb efficiency (η, %) was obtained by the ratio between t_d and t_c. The t_c was the charging time, and t_d suggested the discharging time.

$$\eta = t_d / t_c \times 100 \tag{S2}$$

The relationship between peak current (I) and scan rate (v) in the CV curves can be described as follows:

$$I = av^b \tag{S3}$$

where the b = 0.5 corresponded to the ion intercalation process, and b = 1 implied the capacitance-like behavior.

Capacity contribution can be calculated based on CV results:

$$I\,(V) = k_1 v + k_2 v^{1/2} \tag{S4}$$

where v was the scan rate. k_1v stood for the contribution of capacitive-controlled current, and $k_2v^{1/2}$ reflected the contribution of diffusion-controlled current.[1-2]

The electrochemical impedance spectroscopy (EIS) was performed in a frequency window of 10 mHz to 100 kHz under a voltage amplitude of 5 mV. The diffusion coefficient (D_{Na^+}, $cm^2\ s^{-1}$) was calculated by Equation S5 and S6

$$D_{Na^+} = \frac{0.5R^2\,T^2}{c^2\,F^4 A^2 \sigma^2} \tag{S5}$$

$$Z_{re} = \sigma\,\omega^{-1/2} \tag{S6}$$

where F was the Faraday constant, R was the gas constant, T was the absolute temperature, c was the Na^+ concentration, A was the electrode surface area, σ was the Warburg factor, and Z_{re} was the real part impedance.

Desalination behavior measurements: Charge efficiency (Λ) was quantitatively determined according to Equation S7.

$$\Lambda = F \times \Gamma / \sum \tag{S7}$$

where F ($C\ mol^{-1}$), Γ ($mol\ g^{-1}$), and Σ ($C\ g^{-1}$) depicted Faraday constant, salt adsorption capacity, and charge density, respectively.

The energy consumption (E, $Wh\ g^{-1}$) was obtained as follows.

$$E = v \int idt \,/\, (C_0\text{-}C)\ V \qquad \text{(S8)}$$

where the v indicated a driven potential of 1.2 V, $\int idt$ corresponded to the integrated value of the current transient vs. running time plot, and V (mL) was the rotational solution volume. C_0 and C (mg L^{-1}) were initial and final concentrations, respectively.

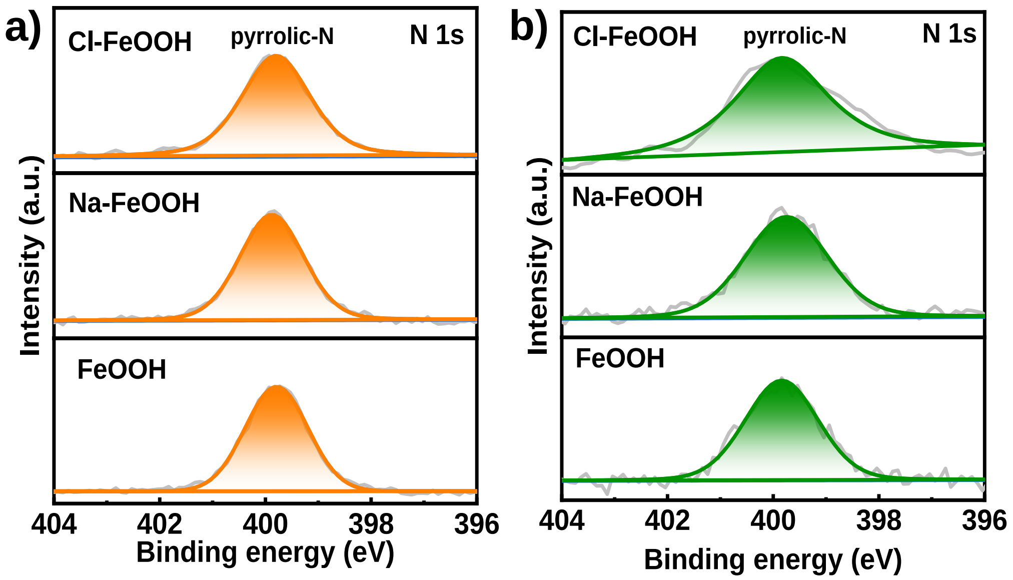

Figure S1. High-resolution N1s XPS spectra of three as-fabricated samples with (a) and without (b) the PVP additive.

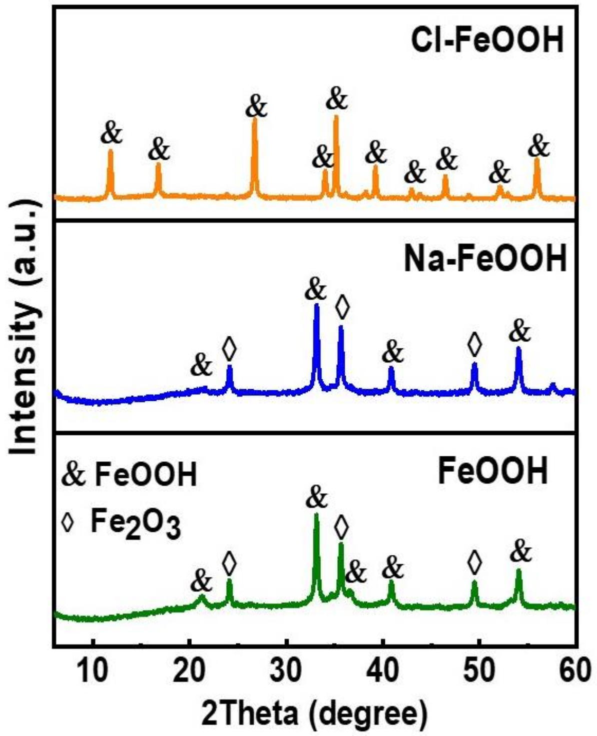

Figure S2. XRD patterns of three as-fabricated samples without the PVP additive.

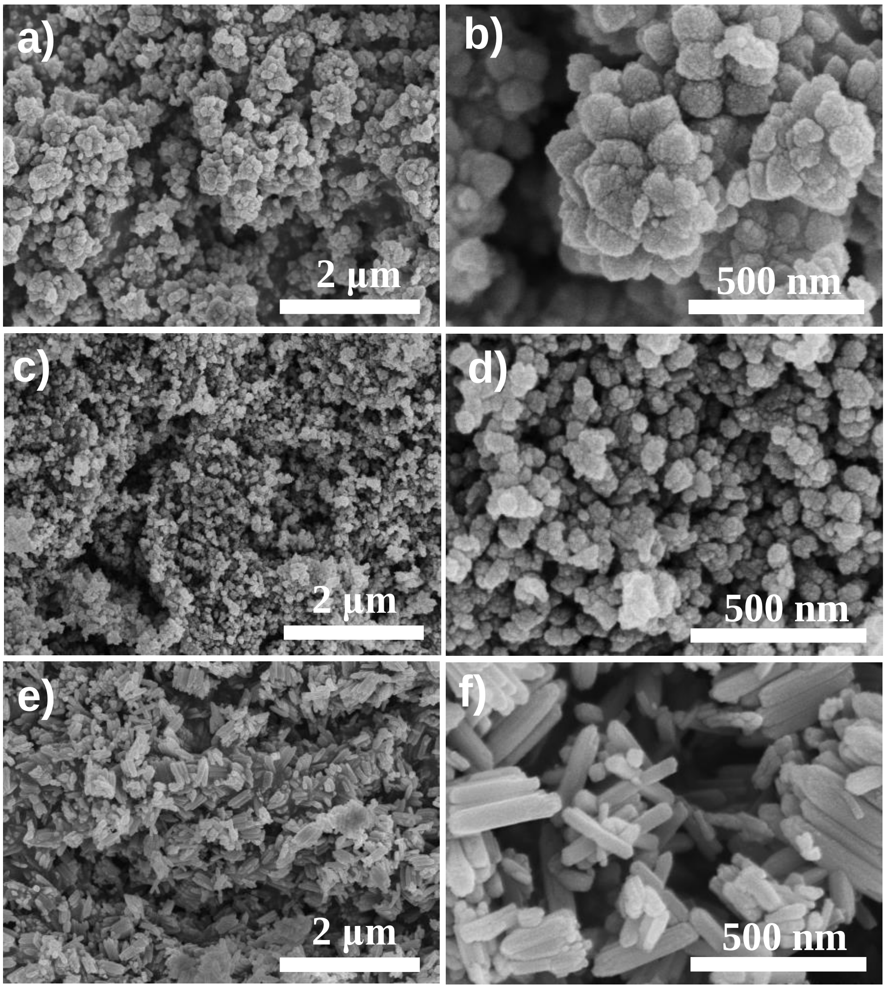

Figure S3. SEM images of three as-fabricated samples without the PVP additive: a,b) bare FeOOH, c,d) Na-FeOOH, and e,f) Cl-FeOOH.

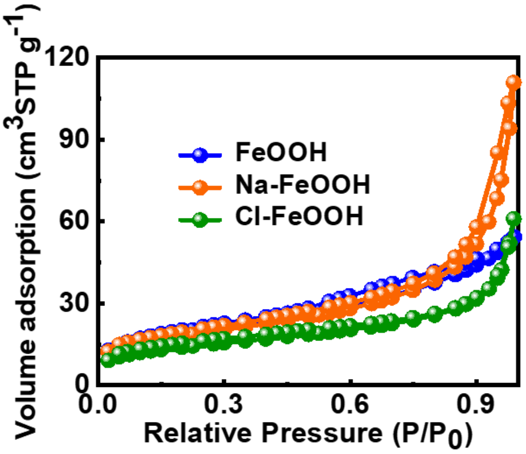

Figure S4. N_2 adsorption-desorption isotherms of bare FeOOH, Na-FeOOH, and Cl-FeOOH.

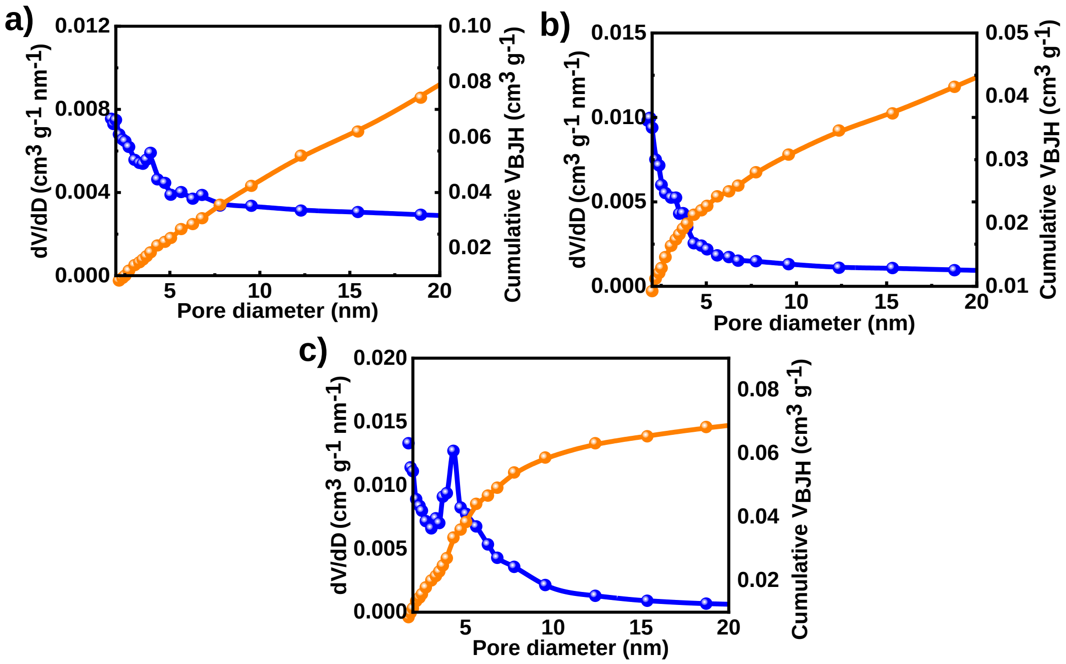

Figure S5. Pore size distribution plots by the BJH method of a) bare FeOOH, b) Na-FeOOH, and c) Cl-FeOOH.

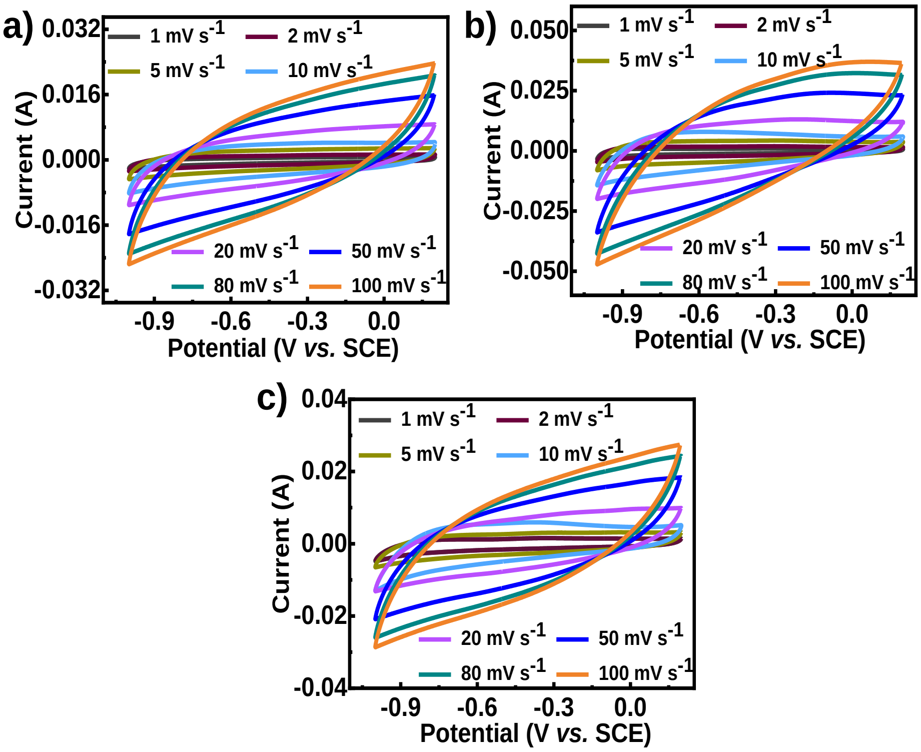

Figure S6. CV curves at different scan rates: a) bare FeOOH, b) Na-FeOOH, and c) Cl-FeOOH.

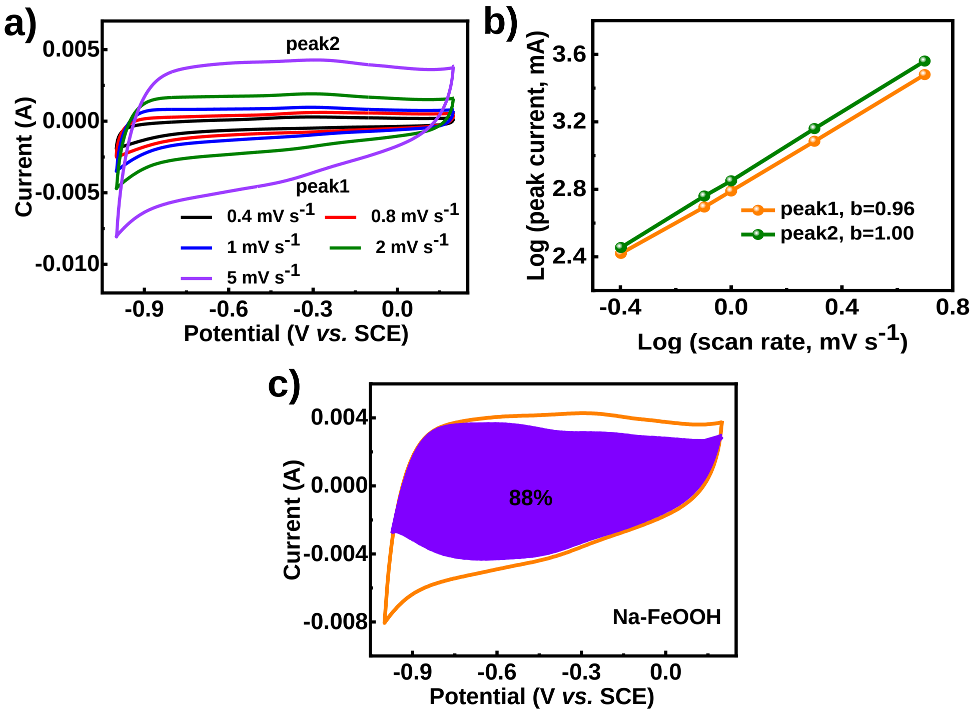

Figure S7. a) CV curves of the Na-FeOOH electrode at various scan rates from 0.4 to 5 mV s^{-1}; b) Log(i_p) vs. Log(v) plots; c) CV curve with the pseudocapacitive contribution fraction (purple region) at scan rate of 5 mV s^{-1}.

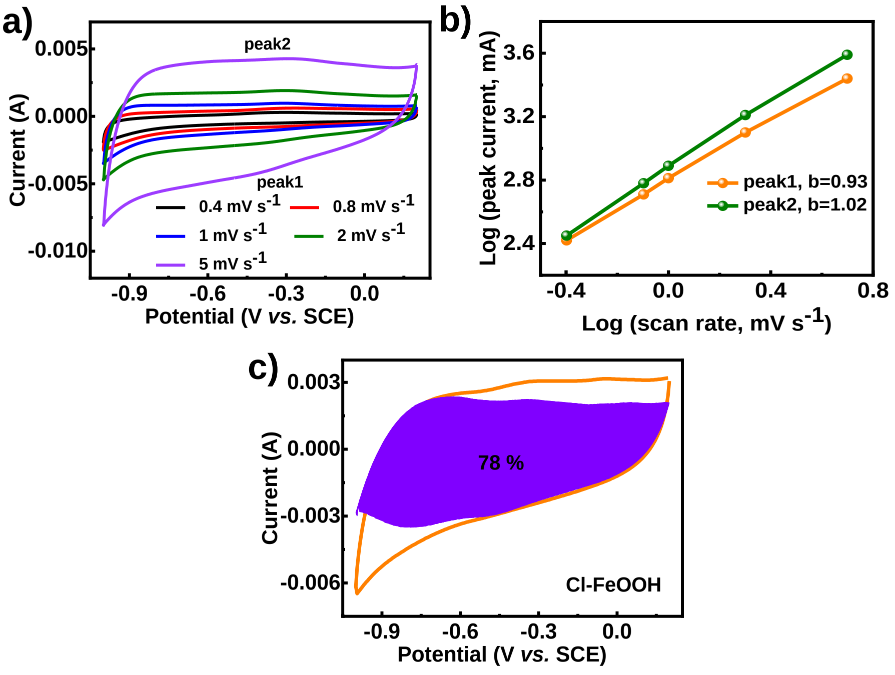

Figure S8. a) CV curves of the Cl-FeOOH electrode at various scan rates from 0.4 to 5 mV s^{-1}; b) Log(*ip*) vs. Log(v) plots; c) CV curve with the pseudocapacitive contribution fraction (purple region) at scan rate of 5 mV s^{-1}.

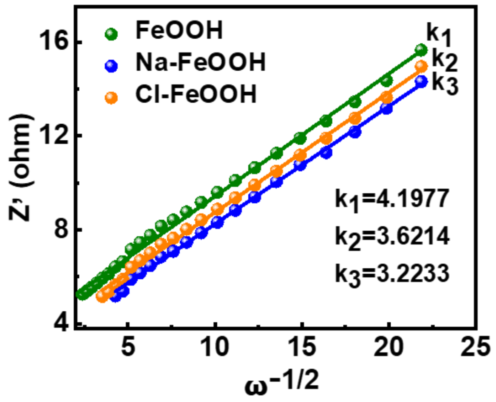

Figure S9. The real part impedance (Z') vs. squared root of the angular frequency ($\omega^{-1/2}$) in Warburg region plots of bare FeOOH, Na-FeOOH, and Cl-FeOOH.

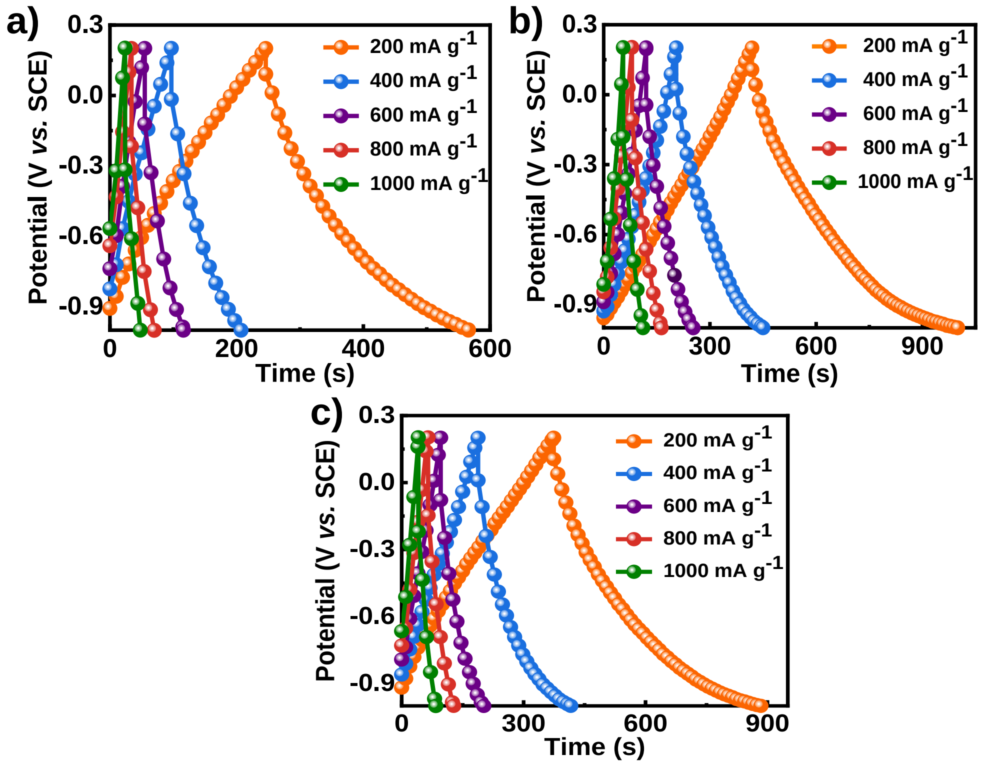

Figure S10. GCD profiles at different current loads: a) bare FeOOH, b) Na-FeOOH, and c) Cl-FeOOH.

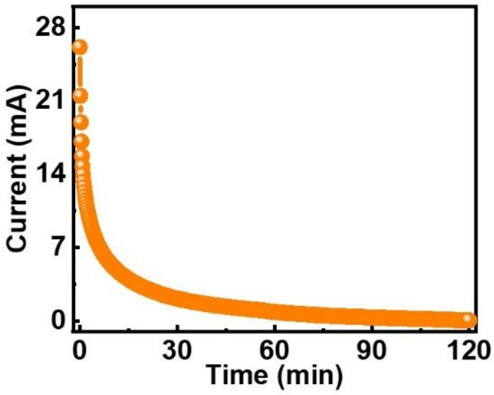

Figure S11. Transient current vs. running time plot of the asymmetric Na-FeOOH//Cl-FeOOH cell during the CDI process.

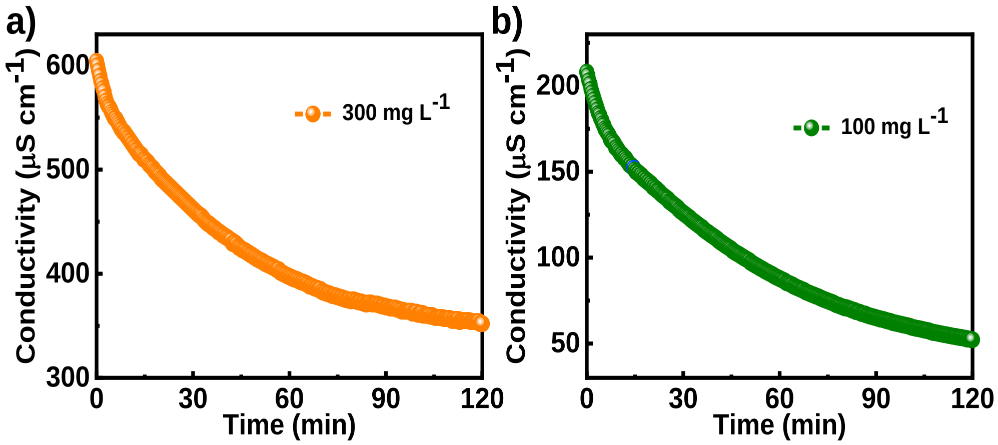

Figure S12. Dynamic solution conductivity vs. running time plots of the asymmetric Na-FeOOH//Cl-FeOOH cell in NaCl solution with different concentrations: a) 300 mg L^{-1} and b) 100 mg L^{-1}.

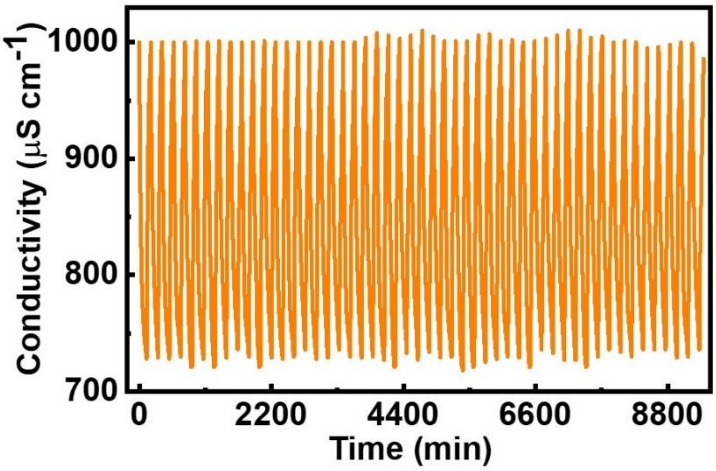

Figure S13. CDI cycling plot of the asymmetric Na-FeOOH//Cl-FeOOH cell.

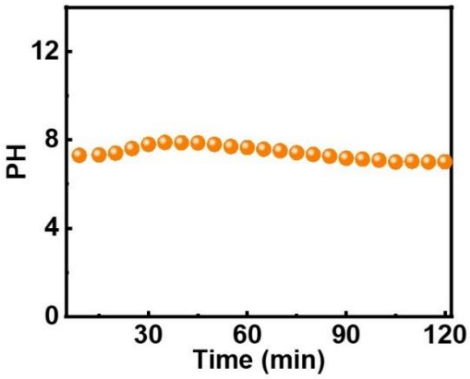

Figure S14. PH vs. running time plot of Na-FeOOH//Cl-FeOOH cell in NaCl solution of 500 mg L^{-1}.

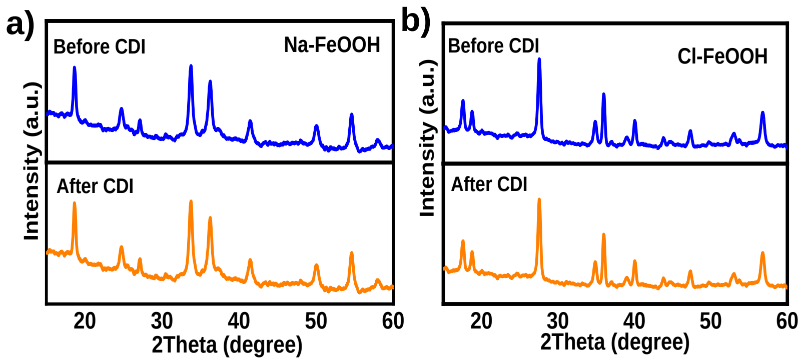

Figure S15. XRD patterns before and after the CDI process: a) Na-FeOOH and b) Cl-FeOOH.

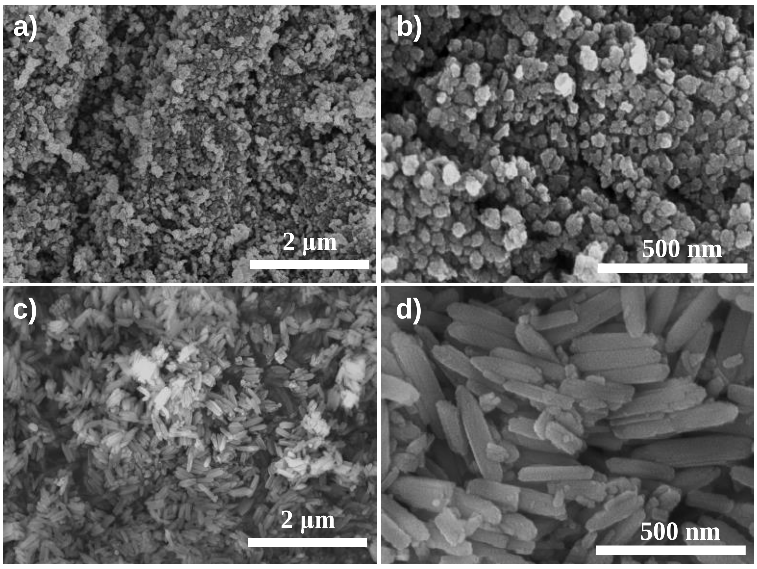

Figure S16. SEM images after the CDI process: a,b) Na-FeOOH and c,d) Cl-FeOOH.

Table S1. Texture properties and element contents determined by XPS and ICP-OES analyzes of three as-prepared samples.

Samples	S_{BET} (m^2 g^{-1})	V_{BJH} (cm^3 g^{-1})	D_{Pore} (nm)	Na content (wt%) (XPS)	Na content (wt%) (ICP-OES)	Cl content (wt%)
Na-FeOOH	64.22	0.17	1.35	0.28	0.24	-
Cl- FeOOH	51.41	0.09	1.35	-	-	4.34
FeOOH	74.87	0.08	1.35	-	-	-

Table S2. Comparative CDI results among different materials.

Samples	NaCl concentration (mg L^{-1})	Voltage (V)	Adsorption capacity (mg g^{-1})	Ref.
P-AC	1000	1.2	22.7	S3
$Li_4Ti_5O_{12}$@C NFAs	100	1.4	25	S4
$Na_{0.71}CoO_2$	500	1.2	34.8	S5
ce-MoS_2	400	1.2	8.81	S6
HC@MnO_2	500	1.2	30.7	S7
VN/NCQDs	500	1.4	23.71	S8
NP-EHPCs	500	1.2	24.14	S9
Na-FeOOH	500	1.2	32.13	This work
Cl-FeOOH	500	1.2	30.22	This work

References

[S1] T. Brezesinski, J. Wang, S. H. Tolbert, B. Dunn, *Nat. Mater.*, **2010**, *9,* 146.

[S2] S. W. Li, Y. C. Liu, X. D. Zhao, Q. Y. Shen, W. Zhao, Q. W. Tan, N. Zhang, P. Li, L. F. Jiao, X. H. Qu, *Adv. Mater.*, **2021**, *33,* 2007480.

[S3] G. Bharath, A. Hai, K. Rambabu, F. Ahmed, A. S. Haidyrah, N. Ahmad, S. W. Hasan, F. Banat, *Environ. Research* **2021**, *197,* 111110.

[S4] L. Guo, D. Z. Kong, M. E. Pam, S. Z. Huang, M. Ding, Y. Shang, C. D. Gu, Y. X. Huang, H. Y. Yang, *J. Mater. Chem. A* **2019**, *7,* 8912.

[S5] Z. Z. Liu, Z. S. Yue, H. B. Li, *Sep. Purif. Technol.*, **2020**, *234*, 116090.

[S6] F. Xing, T. Li, J. Y. Li, H. R. Zhu, N. Wang, X. Cao, *Nano Energy* **2017**, *31,* 590.

[S7] S. Y. Wang, G. Wang, T. T. Wu, C. P. Li, Y. W. Wang, X. Pan, F. Zhan, Y. Q. Zhang, S. F. Wang, J. S. Qiu, *Environ. Sci. Technol.*, **2019**, *53*, 6292.

[S8] J. X. Zhao, Z. B. Zhao, Y. Sun, X. D. Ma, M. D. Ye, X. R. Wen, *Environ. Sci.: Nano* **2021**, *8,* 2059.

[S9] H. Zhang, C. H. Wang, W. X. Zhang, M. Zhang, J. W. Qi, J. S. Qian, X. Y. Sun, B. Yuliarto, J. Na, T. Park, H. G. A. Gomaa, Y. V. Kaneti, J. W. Yi, Y. Yamauchi, J. S. Li, *J. Mater. Chem. A* **2021**, *9*, 12807.
